# Multiparametric prostate MRI quality assessment using a semi-automated PI-QUAL software program

**DOI:** 10.1186/s41747-021-00245-x

**Published:** 2021-11-05

**Authors:** Francesco Giganti, Sydney Lindner, Jonathan W. Piper, Veeru Kasivisvanathan, Mark Emberton, Caroline M. Moore, Clare Allen

**Affiliations:** 1 Department of Radiology, University College London Hospital NHS Foundation Trust, London, UK; 2 Division of Surgery & Interventional Science, University College London, 3rd Floor, Charles Bell House, 43-45 Foley St., W1W 7TS, London, UK; 3 MIM Software Inc., Cleveland, OH USA; 4 Department of Urology, University College London Hospital NHS Foundation Trust, London, UK

**Keywords:** Prostatic neoplasms, Multiparametric magnetic resonance imaging, Quality improvements, Software

## Abstract

The technical requirements for the acquisition of multiparametric magnetic resonance imaging (mpMRI) of the prostate have been clearly outlined in the Prostate Imaging Reporting and Data System (PI-RADS) guidelines, but there is still huge variability in image quality among centres across the world. It has been difficult to quantify what constitutes a good-quality image, and a first attempt to address this matter has been the publication of the Prostate Imaging Quality (PI-QUAL) score and its dedicated scoring sheet. This score includes the assessment of technical parameters that can be obtained from the DICOM files along with a visual evaluation of certain features on prostate MRI (*e.g.*, anatomical structures). We retrospectively analysed the image quality of 10 scans from different vendors and magnets using a semiautomated dedicated PI-QUAL software program and compared the time needed for assessing image quality using two methods (semiautomated assessment *versus* manual filling of the scoring sheet). This semiautomated software is able to assess the technical parameters automatically, but the visual assessment is still performed by the radiologist. There was a significant reduction in the reporting time of prostate mpMRI quality according to PI-QUAL using the dedicated software program compared to manual filling (5′54″ *versus* 7′59″; *p* = 0.005). A semiautomated PI-QUAL software program allows the radiologist to assess the technical details related to the image quality of prostate mpMRI in a quick and reliable manner, allowing clinicians to have more confidence that the quality of mpMRI of the prostate is sufficient to determine patient care.

## Key points

- A semiautomated tool for prostate multiparametric MRI (mpMRI) quality can be built using the Prostate Imaging Quality (PI-QUAL) score.
- Our PI-QUAL software program allows a quick assessment of the technical parameters of prostate mpMRI.
- The application of this tool will help in the future refinement of the PI-QUAL score.

## Background

Image quality plays a fundamental role in multiparametric magnetic resonance imaging (mpMRI) of the prostate; it is essential that the images are of adequate diagnostic quality in order to rule in and rule out clinically significant prostate cancer [1, 2]. In addition to the Prostate Imaging Reporting and Data System (PI-RADS) guidelines v.2.1 [3], which outline the minimum technical requirements (Table 1) and standards for the conduct and reporting of mpMRI of the prostate, there have been two important publications—one from the UK and one from the European Society of Urogenital Radiology (ESUR)/EAU Section of Urologic Imaging (ESUI)—that have stressed the importance of specific quality criteria for the acquisition of prostate mpMRI [4, 5]. In this regard, the recently proposed Prostate Imaging Quality (PI-QUAL) score [6] represents the first attempt to address this issue.

**Table 1:** Minimal technical requirements for multiparametric prostate MRI according to the PI-RADS v. 2.1 guidelines

	Axial T2-weighted imaging (T2-WI)	Diffusion-weighted imaging (DWI)	Dynamic contrast-enhanced (DCE)
**Imaging planes**	The same used for DWI and DCE	The same used for T2-WI and DCE	The same used for T2-WI and DWI
**Slice thickness**	3 mm, no gap	≤ 4 mm, no gap	3 mm, no gap
**Field of view**	12–20 cm (to encompass the entire prostate gland and seminal vesicles)	16–22 cm	12–20 cm (to encompass the entire prostate gland and seminal vesicles)
**In-plane resolution**	≤ 0.7 mm (phase) × ≤ 0.4 mm (frequency)	≤ 2.5 mm (phase and frequency)	≤ 2 mm (phase and frequency)
**Specific recommendations**			
**T2-WI acquisition**	Axial plane: either straight axial to the patient or in an oblique axial plane matching the long axis of the prostate. At least one additional orthogonal plane (sagittal and/or coronal). Three-dimensional axial as an adjunct to two-dimensional acquisitions	–	–
**Low** ***b*** **value**	–	50–100 s/mm^2^	–
**Intermediate** ***b*** **value**	–	800–1,000 s/mm^2^	–
**High** ***b*** **value**	–	Dedicated (≥ 1,400 s/mm^2^) Synthesised (from other *b* values)	–
**Temporal resolution**	–	–	≤ 15 s
**Total observation time**	–	–	> 2 min
**Dose of Gd-based contrast agent**	–	–	0.1 mmol/kg
**Injection rate**	–	–	2–3 cc/s
**Fat suppression and/or subtraction**	–	–	Recommended

**Fig. 1:**
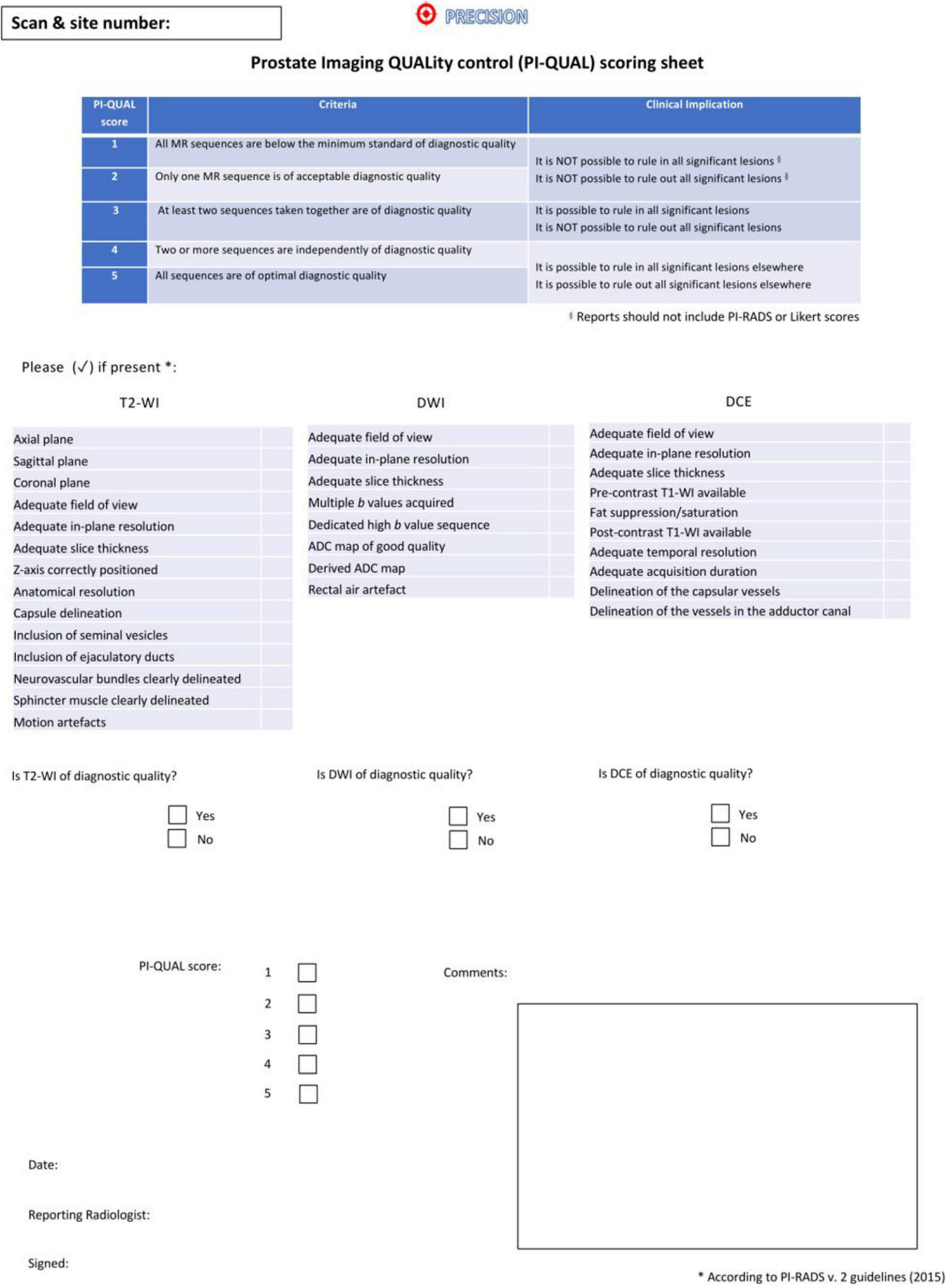
Scoring sheet for assessing the quality of multiparametric magnetic resonance imaging using the PI-QUAL score. ADC, Apparent diffusion coefficient; DCE, Dynamic contrast-enhanced; DWI, Diffusion-weighted imaging; T2-WI, T2-weighted imaging. Reprinted with permission from [6]

This scoring system aims to assess the image quality against a set of predefined criteria (as per PI-RADS guidelines) together with objective criteria obtained from mpMRI of the prostate using a dedicated scoring sheet (Fig. 1). It is based on a 1 to 5 scale where 1 means that none of the three mpMRI sequences (*i.e.*, T2-weighted imaging, diffusion-weighted imaging and dynamic contrast-enhanced sequences) has sufficient diagnostic quality, while a score of 5 means that all of the sequences are of optimal diagnostic quality, and therefore, it is possible to rule in and rule out clinically significant prostate cancer.

As adequate image quality is the prerequisite for a diagnostic scan, it is essential that tools that can help the clinician evaluate the quality of a mpMRI study before reporting are developed. When evaluating the image quality, the manual extraction of technical parameters (*e.g.*, in-plane resolution, field of view or temporal resolution) from the metadata of a mpMRI study requires a significant amount of time, which adds to the time needed for the visual assessment of the anatomical structures and for manually filling in the PI-QUAL scoring sheet.

We developed the dedicated PI-QUAL software program that allows the radiologist to evaluate the quality of mpMRI according to the PI-QUAL scoring sheet using a semiautomated step-by-step workflow. A semiautomated workflow means that some of the steps (*e.g.*, the quantitative extraction of technical parameters from the metadata) are performed by the software while other steps (*e.g.*, the qualitative assessment of specific anatomical structures) are manually inserted by the radiologist.

In this technical note, we discuss this PI-QUAL software program, which we have specifically developed with the intent of assisting the radiologist in daily clinical practice.

## Methods

The PI-QUAL scoring sheet (Fig. 1) was created to standardise the assessment of prostate mpMRI quality and streamline the collection of data both for clinical and research purposes.

All patients included in this report gave written informed consent to have their images used for research and teaching purposes.

### PI-QUAL workflow

After a general window in which the radiologist is asked to enter their name and the name of the scan site (if the program is used for research purposes), the PI-QUAL software automatically extrapolates the technical parameters of T2-weighted imaging, diffusion-weighted imaging and dynamic contrast-enhanced sequences from the raw Digital Imaging and Communications in Medicine (DICOM) images as outlined in the PI-QUAL scoring sheet (Fig. 1) and checks their compliance against the PI-RADS technical requirements. It should be noted that the PI-QUAL software has been built following PI-RADS v. 2.1 guidelines [1].

The radiologist then manually evaluates the scans for the presence (or absence) of the items listed in the ‘visual assessment’ box of the scoring sheet. Finally, the radiologist uses both results to state whether the images for each sequence are of diagnostic quality.

**Fig. 2:**
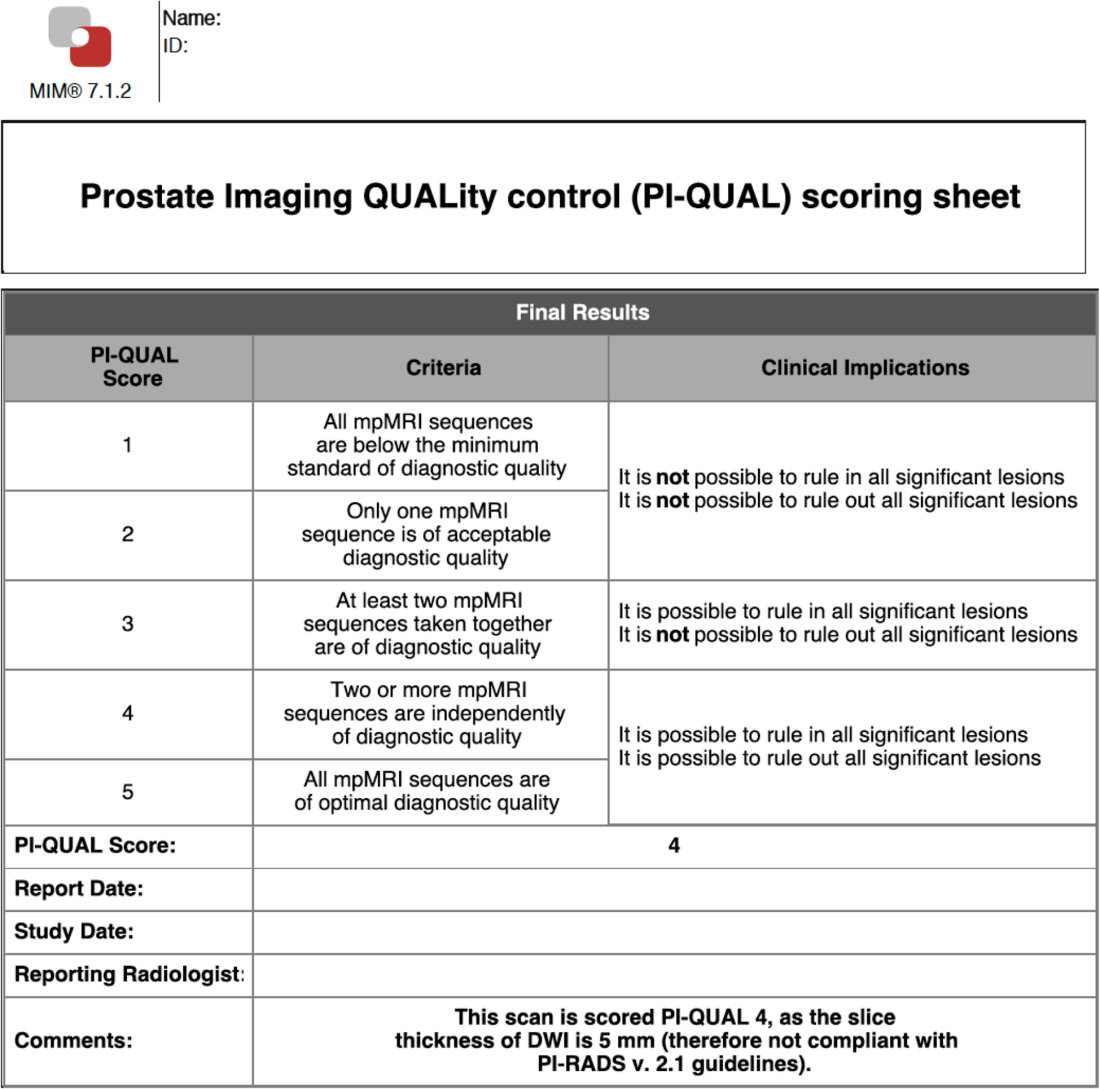
Structured report obtained using the dedicated PI-QUAL software program

**Fig. 3:**
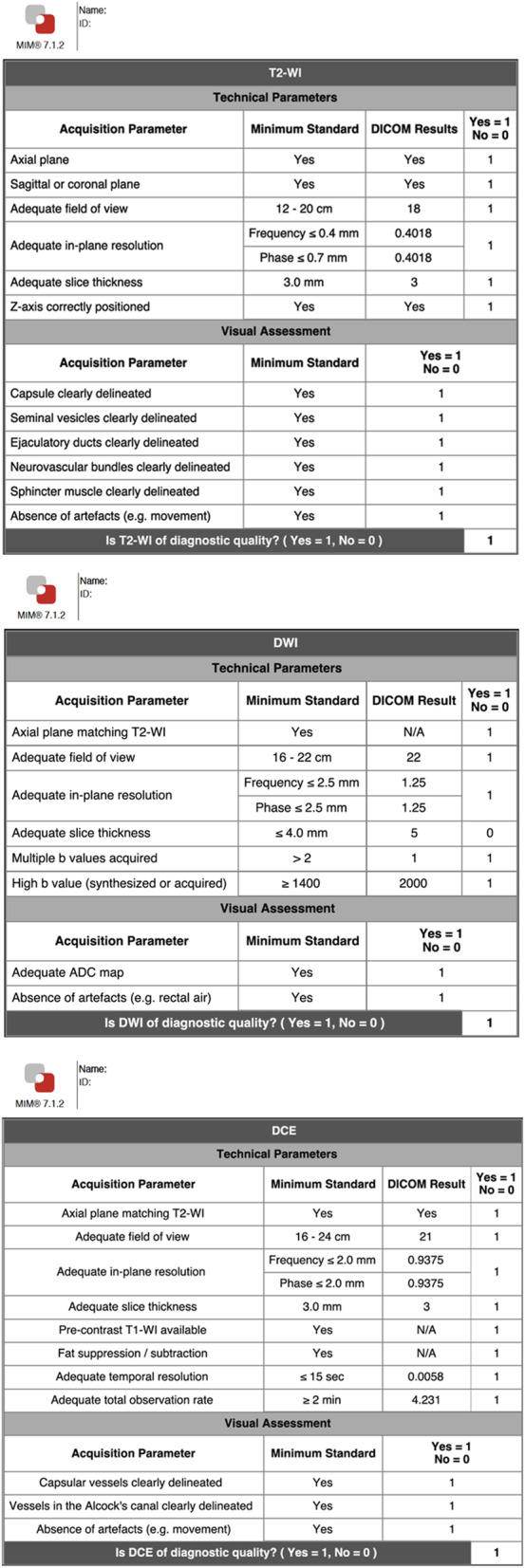
Structured report obtained using the dedicated PI-QUAL software program

**Fig. 4:**
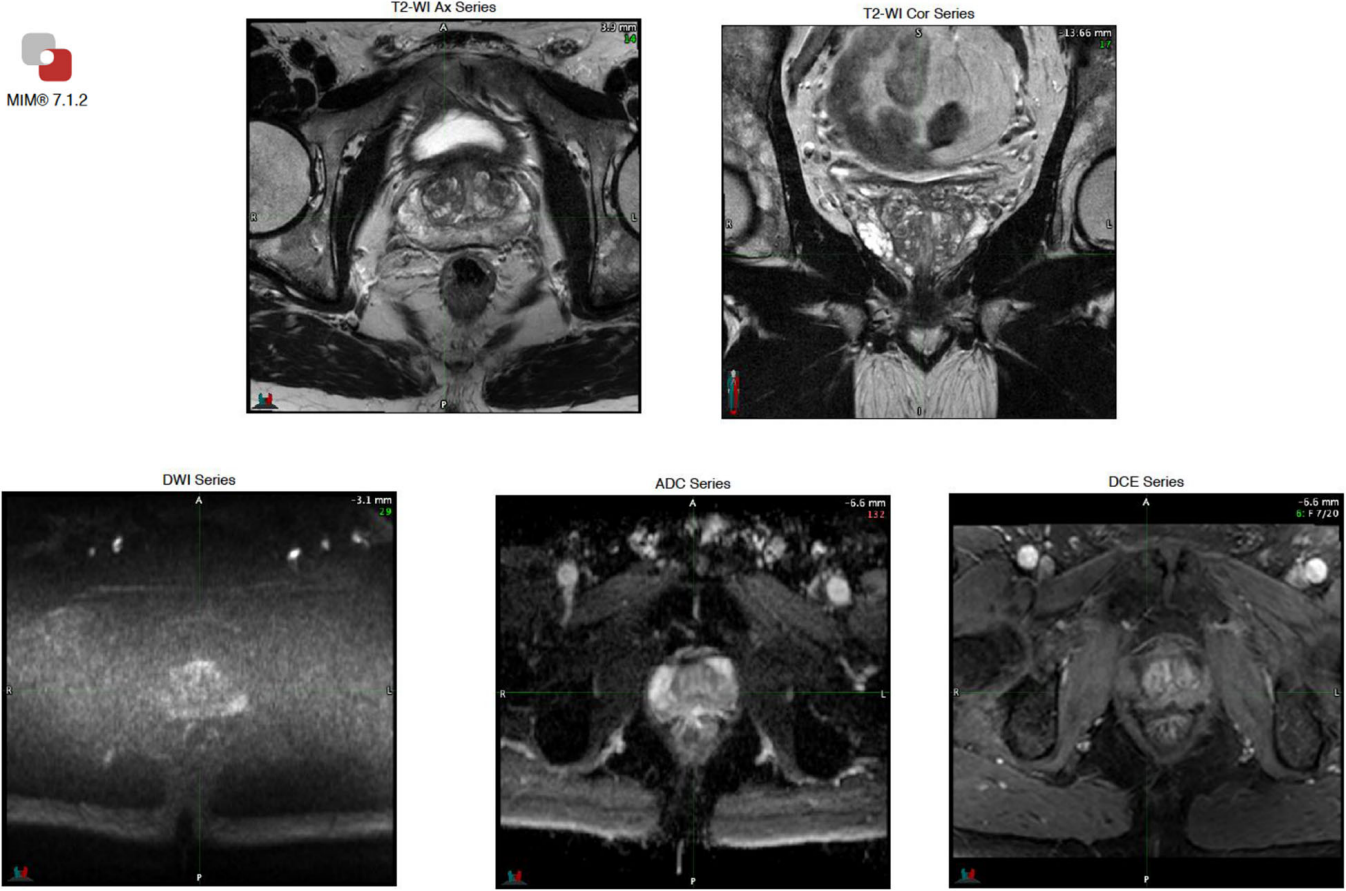
Structured report obtained using the dedicated PI-QUAL software program

In the final step, the operator inserts the PI-QUAL score and has also the possibility to include additional comments and relevant snapshots from the different sequences (Figs. 2, 3 and 4).

### Technical aspects

When the DICOM data is imported, the software parses the header and stores the elements of this information in its metadata repository for the user’s reference when browsing the patient list. The header contents of DICOM datasets are also stored in the memory for retrieval in other software functions. The PI-QUAL workflow is configured to directly read the values of specific tags embedded in the DICOM data from memory storage, then the workflow returns the values of these tags as variables for display to the user or for further manipulation in the workflow.

For example, the slice thickness of the axial T2-weighted image is pulled from the DICOM tag, then the workflow compares this value to the PI-QUAL minimum standard value (as per PI-RADS v. 2.1 guidelines) for slice thickness. The workflow displays a table to the user that contains the image-specific value, the value of the PI-QUAL minimum standard and a boolean pass (yes)/fail (no) result when comparing the image-specific value against the minimum standard value. The workflow gives the user the ability to adjust the pass (yes)/fail (no) results for any DICOM-based technical parameters and perform other visual-based assessments for the image that cannot be directly derived from DICOM information (*e.g.*, clear delineation of certain anatomical structures).

Therefore, the overall quality scores are based upon the radiologists’ interpretations of both the automatic technical parameter results and the visual assessment according to the PI-RADS v. 2.1 guidelines.

### Image analysis

In this study, two specialist consultant radiologists (C.A. and F.G. reporting more than 3,000 and 2,000 prostate MRI scans per year, respectively) analysed in consensus the image quality of 10 multiparametric scans (all without endorectal coil) from different MRI systems and vendors. First, they filled in the PI-QUAL scoring sheet manually and then, after an interval of 8 weeks between the two readings to avoid any recall bias, they re-assessed the image quality using a dedicated PI-QUAL software program (MIM® Symphony Dx v. 7.1.2 - Cleveland, OH, USA).

The time needed to assess the image quality for each scan was recorded for both methods.

### Statistical analysis

Data are presented as medians and interquartile ranges (IQR) and were compared using a two-tailed Wilcoxon test. All statistical analyses were performed by using SPSS (version 27.0; SPSS, Chicago, IL, USA); *p* values were considered to indicate a significant difference when < 0.05.

## Results

Four out of 10 (40%) patients were scanned on a 1.5-T scanner and 6/10 (60%) on a 3-T scanner. In detail, 7/10 (70%) scans were conducted on a Siemens platform (three *Skyra*, two *Verio*, one *Avanto*, and one *Prisma*), 1/10 (10%) on a General Electric platform (*Signa*) and 2/10 (20%) on a Philips platform (one *Ingenia* and one *Intera*).

There was a significant reduction in the reporting time of prostate MR quality using the dedicated PI-QUAL software program compared to the manual filling of the scoring sheet (5′54″ [IQR 5′40″–6′40″] *versus* 7′59″ [IQR 7′26″–8′29″], respectively; *p* = 0.005).

## Discussion

Our study suggests that the use of a semiautomated PI-QUAL software program can be of help to accelerate the assessment of image quality of mpMRI of the prostate.

There has been a lot of interest in the application of artificial intelligence (AI) in radiology over the last few years [7], and currently, there are at least 100 commercially available software products [8]. As far as mpMRI of the prostate is concerned, different AI tools have been developed for a more efficient image interpretation [9–13] but not for the assessment of image quality, which is actually the prerequisite for a correct and reliable image interpretation.

It should be made clear that at present, our PI-QUAL tool is not based upon an AI algorithm, but it is simply a semiautomated software program that combines human and machine steps. However, our PI-QUAL software program could pave the way to the creation of a fully automated AI application by using PI-QUAL evaluations for a large subset of data to train a neural network. This could help assess the image quality during the acquisition of the different prostate mpMRI sequences, for example, recommending a new scan acquisition if the quality is suboptimal.

Since the PI-QUAL score is an aggregate over each modality, two AI approaches would be possible for each sequence. First, given an overall visual assessment, it has been shown to be possible to train an AI to mimic a human grader with no additional information than the visual assessment score [14]. This assessment could replace the entire “visual assessment” ranking for a sequence, or it could be used only on the “absence of artefact” and “adequate ADC map” scores. The second method would apply to the subscores concerning the delineation of various anatomic sub-structures. If a dataset with segmentations could be obtained, a network could be trained both to segment the structure in question and also to express confidence in the segmentation. The usual method would be to train a family of similar networks (*e.g.*, trained on different subsets of the image data or trained with a probabilistic dropout technique) and then compare the segmentations produced. It has been shown that variance in the produced segmentations is a good correlate for the quality of the segmentation [15]. Therefore, experiments would be devised to show that if the network is unable to confidently segment a structure, it is because the structure is not clearly delineated in the image and the poor segmentation is not due to limitations of the segmentation model. If so, this technique could be used to grade the clear delineation of various structures in the T2-weighted and dynamic contrast-enhanced sequences.

In addition, our PI-QUAL tool could contribute to the creation of a cloud-based platform for multiple centres with multiple readers, in order to facilitate the assessment of the inter-reader variability of the PI-QUAL score and also to promote the use of this scoring system (and its future iterations) for clinical and teaching purposes.

There are some limitations to our study. First and foremost, the small sample size (*n* = 10). Second, two radiologists analysed the images in consensus, so we cannot provide the inter-reader variability of the PI-QUAL score in this cohort. However, this has been recently investigated in another cohort, and the results have shown a strong reproducibility in the assessment of PI-QUAL between two expert radiologists [16].

In conclusion, our initial results demonstrate how a semiautomated program can be used to analyse the image quality of prostate mpMRI in a quicker and reliable manner. An assessment of image quality should be performed prior to reporting mpMRI of the prostate so that the clinician and patient can be confident in the result. As stated in the original publication [2], the PI-QUAL score will be refined in the future and an international group is currently working on the next version to see if this scoring system should be still based on a 5-point scale or simplified into a 3-point scale. The application of our tool will help in the future refinement of the PI-QUAL score, and we plan periodic updates of the software and, possibly, the creation of a fully automated AI application.

## Data Availability

Not applicable.

## Abbreviations

AI: Artificial intelligence
DICOM: Imaging and Communications in Medicine
IQR: Interquartile ranges
mpMRI: Multiparametric magnetic resonance imaging
PI-QUAL: Prostate Imaging Quality
PI-RADS: Prostate Imaging Reporting and Data System
